# Editorial: Allergic diseases and neurodevelopment

**DOI:** 10.3389/fped.2023.1199467

**Published:** 2023-04-20

**Authors:** Jennifer K. Straughen, Anita L. Kozyrskyj, Andrea E. Cassidy-Bushrow

**Affiliations:** ^1^ Department of Public Health Sciences, Henry Ford Health, Detroit, MI, United States; ^2^ Department of Obstetrics, Gynecology and Reproductive Biology, College of Human Medicine, Michigan State University, East Lansing, MI, United States; ^3^ Department of Pediatrics, University of Alberta, Edmonton, AB, Canada; ^4^ Department of Pediatrics and Human Development, College of Human Medicine, Michigan State University, East Lansing, MI, United States

**Keywords:** allergic disease, neurodevelopment, attention deficit hyperactivity disorder, tic 11 disorder, immunoglobulin E.

**Editorial on the Research Topic** Allergic diseases and neurodevelopment

Neurodevelopmental disorders, such as attention deficit hyperactivity disorder (ADHD) and autism spectrum disorder (ASD) have risen in prevalence, with global estimates reaching a prevalence of 1% for ASD and a prevalence of 7.2% for ADHD (1, 2). Concurrently, the prevalence of allergic diseases including food allergy and asthma has increased by approximately 50%; this increase comes with a high burden of anxiety and reduced quality of life (3, 4). Bridging these trends are observations made by clinicians and scientists that allergic diseases manifestations, such as atopic dermatitis, allergic rhinitis, food allergy, and asthma, are more common in children with neurodevelopmental disorders than their typically developing counterparts (5–7). It is unknown whether these conditions co-occur due to shared causes or exposures or if the development of one of these conditions increases the risk of the other.

In this special issue on “Allergic Diseases and Neurodevelopment”, weaknesses in previous research on this overall topic are rightly highlighted, foremost being the reliance on data from cross-sectional or case-control studies, which does not allow for evaluation of potential causal mechanisms. The literature review conducted by Chua et al. identified both cross-sectional and longitudinal studies in their summary of the cumulative literature on allergic diseases and neurodevelopment. This review has a special focus on potential biologic mechanisms, including immune dysregulation, epigenetics, and mitochondrial dysfunction that may underlie observed associations between allergic diseases and neurodevelopment. Chang et al. conducted a systematic literature review and meta-analysis, but focused on tic disorders and allergic diseases, finding a correlation between some allergic disease manifestations and tic disorders in children. Differences in the study designs, duration of follow-up time, type of exposure, and outcome measures obtained hindered the interpretation of the findings in both reviews. Few studies have examined how the immune system and neural systems interact to potentially influence the risk of disease throughout the prenatal period and into early life. Additionally, limited research has been conducted to capture biological mediators (e.g., markers of the immune system or alterations in the gut microbiome) that may influence the observed relationships between allergic disease and neurodevelopmental delay. Nonetheless, these combinations of allergic and neurodevelopmental morbidity represent a burdensome phenotype for affected children and families. Epidemiologic research is fundamental to the identification of potential causes and the development of targets for disease prevention.

This special issue references new research findings from two different large birth cohort studies that lend strength to prevailing hypotheses and present opportunities for future research in this field. Interestingly, while both studies broadly suggested that the occurrence of allergic diseases in early life is not associated with later neurodevelopment, subgroup findings in both studies suggest the need to examine these relationships by sex and critical windows of development. The first study by Rodriguez et al. found that immunoglobulin (Ig) E-mediated atopy or food sensitivity at age 1 was not associated with measures of cognition, language, or motor neurodevelopment at 2 years of age. However, boys with atopic or food sensitizations had lower social-emotional development scores. Also using a prospective study design, Straughen et al. found that child IgE measures were not associated with ADHD at 10 years of age. However, maternal prenatal IgE was associated with the development of ADHD at 10 years of age, suggesting that the prenatal period may be an important interventional window. Future work would benefit from investigations into sex-specific effects as there is a growing body of research suggesting that *in utero* exposures may differentially impact the risk of diseases in male and female children (8, 9).

In summary, there remains a need to investigate the co-occurrence of neurodevelopment and allergic diseases, particularly in well-designed longitudinal studies beginning in the prenatal or even pre-conception periods. Few studies have investigated underlying biological mechanisms though several different pathways have been proposed.
